# A Treatise on Human Physiology

**Published:** 1859-03

**Authors:** 


ART. VI.—*A Treatise on Human Physiology. Designed for the Use of Students and Practitioners of Medicine.* By JOHN C. DALTON, JR., M. D., Professor of Physiology and Microscopic Anatomy in the College of Physicians and Surgeons, New York; Member of the New York Academy of Medicine; of the New York Pathological Society; of the American Academy of Arts and Sciences, Boston, Mass.; and of the Biological Department of the Academy of Natural Sciences, of Philadelphia. With two hundred and fifty-four illustrations. Philadelphia: BLANCHARD & LEA. 1859.

Although the mode of investigation of physiological phenomena, and, in fact, much of the general aspect of the science has been materially changed within the last few years, this department of our science has always seemed to us most seductive, whether theorized upon, as by the older physiologists, or studied with the rigorous experimental investigations of those of our own day; and we regardthe scientific life of a man wrapped up in physiological studies, and making advances in the mysterious workings of the animal organism, as one which can leave the most grasping mind nothing to desire. The field is so boundless; the paths which lead from it to kindred studies, so numerous and varied, that occasion is offered for the exercise of almost every quality of the human intellect.

The science of physiology is now most intimately connected with anatomy, chemistry, natural history, and general pathology. Investigations in this direction have, of late, elucidated innumerable disputed points in practical medicine and surgery, and added an interest to obstetrics, which, without this, it could never have offered.

Look back for only a space of thirty years, and see what difficulty the older physiologists experienced, on account of their limited knowledge of minute anatomy. Leeuwenhoec, Muys, Bauer, Fontana, and others, were then applying the microscope to investigations of the tissues, and were publishing most conflicting anatomical descriptions. Take, as an example,the ultimate muscular fibre. In regard to the size, chiefly, these anatomists were at issue. Muys supposed it to be the many hundred times smaller than the finest hair, which is commented upon by Bostock as "too minute to permit us to form any conception of it." Leeuwenhoec stated that they differed in size in different animals, and that the ultimate muscular fibre of the frog were much larger than those of the ox; others thought that it was always of the same size. An hypothesis which was in vogue seventy-five years ago, was, that the muscular fibres were continuous with the nerves. This was supported by no less distinguished a man than Cullen. Boerhaave, Albinus, and other eminent men, thought that the muscles were the same as the tendons, only that the substance of the latter was more condensed. Bauer, however, made some experiments, which were considered very remarkable. These are described in Bostock's Physiology, a work published in 1825: he thought that the muscular fibres were of the same diameter as the blood globules, and succeeded in reducing the fibre to the blood globule, by depriving both of their coloring matter. He then estimated the diameter of both at $\frac{1}{2000}$ of an inch. Bauer evidently saw the little squares which are in the muscular fibrillæ, but he made his estimate much too large, both for these disks and the blood globules; the muscular disks being in reality about $\frac{1}{10000}$ of an inch in diameter, and the blood globule $\frac{1}{3500}$.

Many are, even now, disposed to complain of the indefiniteness of microscopical assertions; but we conceive that the anatomical facts which the microscope has now established, are definite enough, when compared with the blind investigations fifty years ago.

We have mentioned some of the difficulties which were formerly in the way of accurate physiological investigation, when the world was receiving the light thrown upon this subject by Bichat, to give an idea of the necessity of anatomy to the science of life. Animal chemistry was in about the same state; and, it was this state of things which drove thinking minds into the wild theories which have had their rise and fall. Groping in the dark, as they were in that day, appreciating the importance of the knowledge of healthy vital phenomena to the practical branches, it is not to be wondered that men of genius were fain to reason from assumptions, or ill established data, as there were none other at their disposal. Marching hand in hand with these physiological changes, were the exclusive pathological theories. The solidists, the humoralists, the vitalists, the mechanical theories of disease, the chemical explanations of morbid phenomena, the inflammatory theories, the anæmic doctrine, etc., all had their origin from an effort at simplification and generalization; an attempt to bring the pathology of all maladies under one general rule, as it was attempted to make all physiological phenomena amenable to a general law. As science advanced a little, man, in his self-conceit, must give a definite explanation of all the workings of nature; he could not be content to leave anything unaccounted for; and, therefore, the necessity of refuting every theory, which had a shadow of plausibility, swelled the text books on physiology to a most enormous size. Carpenter's Physiology, which is very much used, grew from a book of five hundred and fifty pages, in 1846, to one thousand and fifty in 1853. A work of this size is too much for students, and, indeed, for practitioners. One who makes physiology his specialty finds it useful as containing nearly all opinions upon every physiological subject; but can at once see that it is not the book from which a student is to learn. Students who are learning physiology, and practitioners who wish for facts, without wading through endless discussions, need a small book. Prof. Draper has lately published one of comparatively small size, but this, we fear, does not answer the purpose; it is not a sufficiently good exponent of the present state of physiological science, and much room is taken up with the discussion of questions purely theoretical, and which are not to be settled in that way.

The work before us, however, in our humble judgment, is precisely what it purports to be, and will answer admirably the purpose for which it is intended. It is, *par excellence*, a text-book; and the best text-book in this department that we have ever seen. We have carefully read the book and speak of its merits from a more than cursory perusal. The plan of the work is that adopted by the author in his oral teachings, which is the best arrangement of this subject. In the first place, we have an introduction, containing a few general considerations, no more, indeed, than are absolutely indispensable to a correct understanding of the subject as it is presented in the body of the work. In reading this introduction, we are struck with the vigor and originality of mind which characterize all the author's literary efforts. This forcible grasping of the essence of the subject, without unnecessary prelude or useless expressions, will serve to engage the attention of the student in the important truths on which the science is based. These superadditions are often presented, both in text-books and in the lecture room. A deficiency is especially apt to occur in the description of the organic chemical constituents of of the animal organism; we must not look at fibrin, albumen or casien, as so many atoms of carbon, hydrogen, oxygen, and nitrogen, but merely as *fibrin*, etc. This point is forcibly expressed by the author, who says:

> "It is the object of the anatomist to make himself acquainted with every constituent part of the body. Those parts, therefore, which cannot be recognized by their form and their texture, he distinguishes by their chemical reäctions. But afterward, he has no occasion to decompose them further, or to make them enter into new combinations; for he only wishes to know these substances *as they exist in the body*, and not as they exist under other conditions."

This is the great point, namely, to know these substances "*as they exist in the body*." It does not convey any very distinct idea to say that albumen is composed of C. H. N. O. S. Ph. in certain properties, for when we look at fibrin, which possesses proporties widely different, no difference in the quantity of these elements can be detected by the chemist; and the chemist, from these elements, cannot reconstruct albumen or fibrin. This is the chemical division of anatomy; and we employ this to ascertain the chemical properties of these substances, not to decompose them, for, as the author states, we only wish to study them in their actual condition in the body.

The author is equally happy in his enunciation of a principle, which should regulate our study of physiological phenomena. We must not, he says, make phenomena correspond to laws, or neglect a phenomenon because it is not in exact accordance with some so-called law. "For the law is not superior to the phenomenon, but on the contrary, depends upon it, and derives its whole authority from it." This should be the great starting point for all scientific investigations; and with workers who regarded this precept, nothing would be lost, no observations would be useless; but, as it is, we need not say how often phenomena are innocently perverted or neglected, because they do not conform to some law, or even to a preconceived notion. This is, perhaps, more liable to occur in microscopic researches than in any other, for, with this instrument, it is easy enough to see, or imagine to see, almost anything. This introduction occupies but ten pages, but it contains a great deal in that small space, giving an earnest of the manner in which questions will be considered in the body of the book.

The author has divided the study of physiology into three sections. The first treats of nutrition, in all its bearings. The second treats of the nervous system; and the third, of reproduction or generation.

The section devoted to nutrition is, by far, the most elaborate and best digested part of the work. The importance of this subject, in some of its bearings, its relations to practical medicine and pathology, and the brilliant results of investigations which have lately been made in this direction in all parts of the scientific world, have rendered it now, perhaps, the most interesting division of physiology. In this department, the author has distinguished himself both as a writer and teacher, and we venture to hope that America will yet have a considerable share, through Prof. Dalton, in unraveling some of the points which are yet *sub judice.* The author first describes the proximate principles, as they are called by Robin and Verdeil. These he defines in the following way: "A proximate principle is properly defined to be *any substance, whether simple or compound, chemically speaking, which exists, under its own form, in the animal solid or fluid,* and which can be extracted by means which do not alter or destroy its chemical properties." These he then divides into three classes: First, those which are purely *inorganic* in their nature. Second, *crystalizable substances of organic origin.* Third, *organic substances proper.* In the first class, are included those substances which are found in, and derived from, the external world. In the second class, are included those which make their appearance first in the organic world, and which are crystallizable having a definite chemical composition: as the sugars, and fats. In the third class, are included those which appear first in the animal organism, and which do not have a definite chemical composition; the protein compounds are examples of this class. After a consideration of the particular substances belonging to these classes, with an account of the method of separating them from each other, or their chemical anatomy, we have, following in natural order, the substances by which these are renewed or regenerated in the processes of nutrition, or articles of food. After having considered these substances, we have an account of the processes by which the more complex are prepared for the nourishment of the tissues, namely, digestion.

In following our author through the consideration of the processes of digestion, we find nothing which need detain us in the physiology of mastication, etc., and we come to a consideration of the digestive fluids. The idea that the first of these fluids, the saliva, had a certain action upon the amylaceous articles of food, received much apparent support from the fact, that starch was converted into sugar, by the saliva, in ten minutes, or less, when exposed in a test-tube to a temperature of 100 degrees. Experiments which are cited by Dr. Dalton, however, and which he has himself repeated, show conclusively that, though this action undoubtedly takes place in the test tube of the chemist, it does not result, in the carnivora, in the laboratory of nature. If a dog be fed with starch, and the fluids be drawn off from the stomach, by means of a gastric fistule, it can be detected by the iodine test, for ten, fifteen and twenty minutes after, as starch, and is never changed into sugar in that situation. This fact Dr. Dalton attributes to the admixture of the gastric juice, which prevents the effect which is produced by the saliva in the test-tube. The function of the saliva, then, is purely mechanical; and has not the chemical action which is super-added to this by some physiologists. The mechanical action of the saliva is an established physiological fact, the only disputed point being its action or non-action, in the normal digestive processes, upon starch.

Following, then, the alimentary mass into the stomach, we come to the consideration of the "gastric juice and stomach digestion." After a clear and graphic description of the mucous membrane of the stomach, the author, passing over the numerous theories, which were formerly in vogue in regard to the action of the stomach, commences with the experiments upon the celebrated Alexis St. Martin, the Canadian boatman, in whom there existed a gastric fistula. Here, in reality, we have the commencement of a proper understanding of the action of the stomach and gastric juice, which has been much advanced by recent experiments upon inferior animals with artificial fistulæ. One of the great benefits which resulted from experiments upon St. Martin, was, that we were shown the value of similar experiments upon the lower animals. Undoubtedly, the experiments which have been made upon dogs with gastric fistulæ would have been made if the case of St. Martin had never occurred; but we would then have been in uncertainty in regard to the analogy which exists between the stomach digestion in dogs and the human race. In regard to the chemical properties of the gastric juice, it was not formerly acknowledged that its reaction was invariably acid; but now this point is definitely settled, and the division of opinion among physiologists of the present day is in regard to the kind of acid which is present. This free acid is now known to be a most important element, and, indeed, indispensable to the solvent property of the fluid. Opinions upon this subject are divided between three substances: the muriatic acid, the lactic acid, and the biphosphate of lime. The latter hypothesis is supported by a French observer, M. Blondlot, who supports his theory in a late number of the *Journal de la Physiologie.* His arguments, however, do not appear to us to be very conclusive. Dr. Dalton is in favor of the lactic acid, which is the opinion of Prof. F. G. Smith, of Philadelphia, MM. Robin and Verdeil, Bernard, and most French physiologists. Bidder and Schmidt, however, and Prof. Dunglison assert that they have always found, in the gastric juice, an abundance of the chlorohydric acid. Dr. Dalton refers to a series of experiments by Bernard, which have shown that the hydrochloric acid, which was detected in these experiments, probably resulted from a decomposition of the chlorides, and that the lactic acid is the free acid, at least in the gastric juice of the dog.

The next in the order of digestive fluids is the intestinal juice. Of this fluid our information is but meagre: it has been found exceedingly difficult, and, indeed, almost impossible, to obtain it in its pure state in sufficient quantity for purposes of study: the author, with commendable judgment, has, in this instance, as in other cases, where the physiology was a matter of speculation, refrained from profitless discussion. The space devoted to the consideration of the intestinal juice does not occupy more than three pages. He shows, however, that this fluid, whatever may be its chemical properties, has a decided and rapid action upon the amylaceous articles of food; a dog which has been fed largely with meat and boiled starch, disposing of all of the latter article in an hour's time.

The emulsifying properties of the pancreatic juice, as demonstrated by Bernard, are next considered; following which, is an account of some instructive experiments by the author, upon intestinal digestion. This subject has been little studied by physiologists; and since the peculiar, and almost exclusive actions of the various digestive fluids upon different elements of food has been demonstrated, as the action of the gastric juice upon the albumenoids, leaving the other elements untouched, dissecting, for example, the areolar tissue from between the fat cells, the action of the pancreatic juice upon the fats, or that of the intestinal fluid upon starch, these functions have been considered rather too exclusive. The gastric juice is generally thought to finish its digestive function in the organ in which it is secreted; but Dr. Dalton has shown, from examinations of the contents of the small intestines, drawn from an artificial intestinal fistula, that the solvent action of the gastric juice operates after the alimentary substances after they pass out at the pylorus. A microscopical examination of the muscular fibre, as it passes down the alimentary canal, shows a progression of the process of disintegration; its anatomical characteristics becoming less and less marked as it descends. In the next chapter, which treats of absorption, we find nothing which attracts our attention as particularly new.

We now come to the consideration of that great organ, which was formerly accused of being the cause of so many disorders, namely, the liver. When we glance for a moment at the physiology of the bile, we will see how vague must have been the pathology of this fluid, when we knew even less of its *rôle* in the animal economy than we do at the present day. Even now it is not uncommon to find physicians who attribute all obscure disorders to the liver; "liver complaint" is still prevalent; and almost every one is "bilious" at a certain time of the year. But let us look at the actual state of our knowledge in regard to the function of the bile, which is admirably expressed in the book before us, and see its bearing upon the pathology of the liver. We do not certainly know whether this fluid is excrementitious, or whether it plays an active part in the economy; physiologists are divided upon this point; and the arguments which would lead us to the first supposition are met by others, which point decidedly to the second. The pathology of a part must have physiology for its basis, and we cannot say that the function of an organ is disordered, when we do not know the nature of that function.

We have examples of advances in the physiology of organs, which are not accompanied by an equal march in pathology, and in none is this more strikingly shown than in diabetes mellitus, and its relation to the newly discovered sugar producing function of the liver. At the first blush, it would seem that we had made a great advance in the pathology of diabetes, when we discovered where sugar was produced in the animal economy, and where it was destroyed; but we cannot now say that our pathological knowledge of this disease has become much more certain, or that our treatment is less blind or empirical. In regard to the glycogenic function of the liver, which was discovered by Bernard in 1848, it is a curious fact that from time to time, it has been absolutely denied, and experiments have been made, tending to show that the liver has no such office; we cannot conceive how any one, who has studied the experiments of Bernard, can have the slightest doubt as to the facts which they established.

We next have a short chapter on the spleen, long enough, however, to give us all that is known of the function of this gland. Though we were enabled, by the researches of Bernard into the glycogenic function of the liver, to view this organ as a ductless gland as well as an ordinary gland, in view of its secretion of bile, very little advance has been made in our knowledge of the other ductless, or blood glands, like the spleen, or thyroid gland. Passing over the numerous hypotheses in regard to the spleen, Dr. Dalton is disposed to regard its function as connected with that of the abdominal lymphatic glands; he considers the spleen "as an unusually developed lymphatic, or mesenteric gland."

The remaining chapters of section first are devoted to the blood, respiration, animal heat, circulation, and secretion. It is not our purpose to follow the author through his consideration of all these subjects; some of the disputed points only, and but few of these, will engage our attention.

In reading the chapter on respiration, we were disappointed in not finding more definite statements in regard to the relative quantities of oxygen and carbonic acid in the arterial and venous blood. Magnus, whose tables are quoted by every author on physiology, is here referred to, in regard to the proportion of oxygen to carbonic acid in arterial and venous blood. It is an important point, however, to determine whether there is more carbonic acid in venous or arterial blood. By almost all authors, Magnus is quoted as giving more carbonic acid to the venous blood; but Robin and Verdeil quoting directly from the work of Magnus, say that there is *actually more carbonic acid in the arterial than in the venous blood.*

"The carbonic acid contained in the blood would occupy, in a gaseous state, a space varying from a third to a fifth of that occupied by that liquid. After Magnus, there is more of it in the arterial blood than in the venous blood, in the proportion of 6.49 cubic *centimètres* in 100 in the arterial blood, to 5.50 cubic *centimètres* in the venous blood, that is to say, in the proportion of 0.99 gram, oi one-fifth in favor of the arterial blood, which gives 0.123 grams in 100 in the arterial blood, and 0.104 grams in the second. As already, there is more oxygen in the proportion of 2.41 or 3 to 1.20, and of azote in the proportion of 1.51 to 1.00, we see that there is more gas in the arterial blood than in the venous blood."

This is a literal translation from the work of Robin and Verdeil, and the figures are deduced from the tables of Magnus, which are there quoted from his original work. We do not propose to go into the physiological significance of these facts, but have quoted them as in opposition to the assertion of nearly all physiological writers, who generally quote Magnus, as showing exactly the reverse. We were surprised that Dr. Dalton, in referring to the work of Robin and Verdeil, did not give attention to this point.

Passing now to circulation, we find an important point of issue in the theory of the production of the sounds of the heart. The mechanism by which the second sound is produced, is now definitely settled; but that of the first sound is still under discussion. The view of Dr. Dalton, in reference to the production of the first sound, is undoubtedly correct as far as it goes, but the observation of Prof. Austin Flint upon this point, which appeared but a few weeks ago in the transactions of the American Medical Association, seem to throw more light upon this subject. According to Dr. Dalton, the first sound is "dependent *altogether* upon the closure of the auriculo-ventricular valves," as the second sound is experimentally demonstrated to be dependent upon the closure of the aortic and pulmonary valves. There is no doubt, in our mind, that the closure of the auriculo-venticular valves is *one* of the means of production of the first sound; and the anatomy of these valves (their greater length) accounts sufficiently for the difference in pitch between the two sounds, but it is not sufficient to account for the great difference in duration. Prof. Flint, by a series of observations upon the healthy chest, has resolved the first sound into two elements, a valvular element, which alone exists in the second sound, and an element of impulsion: he is able to separate these two elements by ausculting in the erect and supine position, the latter removing the heart from the thoracic parietes, or by interposing some soft substance, like a few folds of a handkerchief, between the pectoral extremity of the stethescope and the chest. In this way, when the impulsion element of the first sound is eliminated, the first sound is of the same duration as the second, but invariably lower in pitch; the first sound, also, has the valular quality, which is so marked in the second. These observations appeared while Dr. Dalton's work was in press. The movements of the heart are very accurately described, and agree with the first description, as given by Harvey. At each contraction, the heart elongates, hardens, the apex is pushed forward, it moves slightly from left to right, and rotates from left to right. These movements are described differently by some authors; but a careful examination of the heart, exposed in a living animal, will demonstrate them.

A consideration of the different secretions finishes section first, and the rest of the work is devoted to the nervous system and reproduction.

We have already occupied as much space in this review as the plan of our journal permits us to devote to a single work, and we had intended, indeed, to devote most of our attention to the chapters upon nutrition, because here the author is particularly clear and original; the statements which he has here made are most of them deduced from experiments, which are generally described, and can be repeated by those interested in this subject. The physiology of the nervous system is by no means as interesting as that which we have just finished; and the author is evidently of our opinion, for here he is much less elaborate. We have, however, in this section, the subject presented in a clear and forcible manner, and an account of most of the experiments which have been made in this department, and which have developed important facts. But, passing to reproduction, the interest with which we studied the processes of nutrition in section first is revived. We can forgive the author for being a little meagre in section second, for the subject itself is barren; there is, also, a cruelty in the experiments on this subject, which makes a person of tender sensibilities unwilling to pursue it by means of vivisections.

The author commences the section on reproduction, with a consideration of its nature, and a description of the different modes by which it is accomplished; then follows a discussion of the subject of spontaneous generation; an idea which is now almost universally abandoned. We then come to "sexual generation," and the mode of its accomplishment. Following the processes of sexual generation in plants, and in the lower orders of animals, we finally come to the generation in those of a higher grade, the anatomy and production of the egg by the ovaries in different classes of animals, with a similar account of the male organs of generation and their product Rising in the scale of being, we see the important bearing of the corpus luteum, and the changes impressed upon it by the fecundation or non-fecundation of the ovum; in other words, the corpus luteum of menstruation and pregnancy. The investigations into this subject, in which the differences between the corpus luteum of menstruation and of pregnancy were so clearly established, are sufficiently familiar to the profession, as forming the basis of an essay, to which the prize of the American Medical Association was awarded some years since.

The section on generation is, throughout, as complete as could be desired, and is the best digest of the subject we have ever seen. We were especially struck with the author's description of the anatomy of the placenta; having in view the varied and obscure description which we have of this organ in most text-books on physiology, we studied Dr. Dalton's account with a great deal of attention, and are convinced that no student can peruse that chapter, without having a perfectly clear idea of the subject. The anatomical view which Dr. Dalton adopts is experimentally proven by him, and leaves no room for doubt, as to its reliability. The fœtal tufts, which are the enlarged villi of the chorion, during the third month, have considerably enlarged, and become branched in the uterine follicles; this enlargement continues until the capillaries surrounding the uterine follicles become fused together, and form sinuses, which receive the enlarged tufts of the chorion, and each enlarging in opposite directions, the walls of the sinuses and the walls of the tufts become adherent, so that it is impossible to separate them without rupture. There is, however, no absolute connection between the two systems of vessels; the processes of interchange between the mother and the fœtus, which are so indispensable to the development of the latter, go on, by endosmosis, through an intervening membrane.

The fact that there is no connexion between the maternal and fœtal uterine vessels, is capable of demonstration if we can obtain the uterus of an undelivered woman at full term. One author has had four opportunities of this kind, and has demonstrated the facts which we have just stated. In these experiments, he removed the uterus from the woman, and found the placenta still firmly attached.

"Let the fœtus now be removed, by dividing the umbilical cord, and the uterus, with the placenta attached, placed under water, with its internal surface uppermost. If the end of a blowpipe be now introduced into one of the divided vessels of the uterine walls, and the air forced in by gentle insufflation, we can easily inflate, first, the venous sinuses of the uterus itself, and next, the deeper portions of the placenta; and lastly, the bubbles of air insinuate themselves everywhere between the fœtal tufts, and appear in the most superficial portions of the placenta, immediately underneath the transparent chorion; thus showing that the placental sinuses which freely communicate with the uterine vessels, really occupy the entire thickness of the placenta, and are equaly extensive with the tufts of the chorion."

These experiments demonstrate, beyond a doubt, the extent of the uterine sinuses, and the fact that there is no direct communication between the blood of the mother and the blood of the fœtus.

The remainder of this section is devoted to involution of the uterus and the processes of development of the embryo. This part of the subject is extremely interesting, but we have not the space to extend our analysis.

Looking back upon the work we have just finished, we must say a word concerning the excellence of its illustrations. No department is so dependent upon good illustrations, and those which keep pace with our knowledge of the subject, as that of physiology. The wood cuts in the work before us are the best we have ever seen, and, being original, serve to illustrate precisely what is desired. Authors have been in the habit of drawing too exclusively upon the mass of wood cuts which are in the possession of medical publishers; cuts must be original, to illustrate to the entire satisfaction of the author, unless his work be but the merest compilation. There is no such objection to be made to the work before us; for of two hundred and fifty-four illustrations, two hundred and forty-three are engraved from the original drawings of the author, and many of them are more graphic than any we have ever seen. As the author says in his preface, in the departments of the nervous system and reproduction "simple, clear, and faithful illustrations are indispensable for the proper understanding of the printed description," and in this he has most admirably succeeded.

